# Emergence of Clinically Relevant Non-Tuberculous Mycobacterial Infections in Saudi Arabia

**DOI:** 10.1371/journal.pntd.0002234

**Published:** 2013-05-30

**Authors:** Bright Varghese, Ziad Memish, Naila Abuljadayel, Raafat Al-Hakeem, Fahad Alrabiah, Sahal Abdulaziz Al-Hajoj

**Affiliations:** 1 Mycobacteriology Research Section, Department of Infection and Immunity, King Faisal Specialist Hospital and Research Centre, Riyadh, Saudi Arabia; 2 Ministry of Health, Riyadh, Saudi Arabia; 3 Department of Medicine, King Faisal Specialist Hospital and Research Centre, Riyadh, Saudi Arabia; University of Tennessee, United States of America

## Abstract

**Background:**

Non-Tuberculous Mycobacteria (NTM) are emerging around the world due to a higher prevalence of immunosuppressive illness and therapy. Saudi Arabia is not an exception as there have been novel mycobacterial species also identified. In addition, several published case reports from different parts of the country suggest a growing pathogenic potential of NTM. As the first nationwide study, we sought to gain an insight into the species diversity of NTM clinical isolates.

**Methodology/Principal findings:**

During June 2009–July 2010, 95 clinical isolates were collected from tuberculosis reference laboratories in major provinces within Saudi Arabia and subjected to standard line probe assay techniques to identify their species. Diagnostic guidelines of the American Thoracic Society were applied to determine the clinical relevance of respiratory isolates.

Species diversity (13 species) was very high and dominated (61.0%) by rapid growing NTM. The major species obtained were *Mycobacterium abscessus*, *M. fortuitum, M. intracellulare* followed by *M. kansassi, M. gordanae* and *M. avium*. Interestingly this study reports for the first time the clinical relevance of *M. celatum, M. xenopi, M. scrofulceum, M. lentiflavum, M. asiaticum* and *M. simiae* in Saudi Arabia. Of the total, 67.1% were clinically relevant respiratory cases, 23.2% were non-respiratory cases and 9.7% were respiratory colonizers. Coexisting illness was reported in 53.7% of the studied cases. The major risk factors observed among the patients were previous history of tuberculosis, chronic obstructive pulmonary disorder and human immunodeficiency virus infection.

**Conclusion/Significance:**

The high rates of clinically confirmed respiratory cases suggest that NTM infections are indeed a new challenge to health authorities. The current findings show an opposite picture of the Western world where *M. avium complex* and particularly slow growing NTM are the most predominant respiratory pathogens. The complexity of species demands an immediate strengthening of the current diagnostic facilities.

## Author Summary

Non-Tuberculous Mycobacteria (NTM) are opportunistic human pathogens. Global reports show an emergence of various NTM diseases among immuno-suppressed as well as immuno-competent individuals. In Saudi Arabia, data are scarce on the prevalence of NTM and their genetic diversity. Currently, NTM infections are neglected in the country and the real magnitude of problems is still masked. As the first study of its type to be conducted in Saudi Arabia, we sought to explore the species spectrum of NTM by utilizing a nationwide collection of clinical isolates. The NTM species diversity is large and shows a different trend compared to Western countries as rapidly growing mycobacterial species are the most prevalent to cause diseases in the country. A large number of clinically relevant pulmonary cases show high pathogenic potential of NTM. Indeed, timely identification of mycobacterial species will lead to adapting an appropriate therapy and supports the implementation of control policies which is urgently needed in the country.

## Introduction

Globally humans are exposed extensively to environmental sources (soil, water, plant and animal materials) of mycobacteria. Predominantly Non-Tuberculous Mycobacterial (NTM) disease develops among immuno-compromised individuals although recently they emerge among immuno-competent personals also [1]. Generally NTM are resistant to most of the disinfectants and when present on non-sterile patients samples and contaminated medical equipment leads to pseudo outbreaks [2]. The international guidelines to establish the pseudo-infection from NTM disease and their clinical relevance has been published by the American Thoracic Society (ATS) and Infectious Disease Society of America (IDSA). These guidelines recommends minimum of two separately expectorated sputum samples or one bronchial wash or lavage culture positivity in patients with pulmonary symptoms and suggestive chest radiograph images to confirm a relevant pulmonary infection. However culture from any sterile site can be considered as part of a disease [1]. Indeed it is crucial to detect and identify the NTM up to species level rapidly during early stages of diagnosis. Rapid diagnosis supports the infection control and provision for appropriate drug regimens though it differs from tuberculosis treatment.

Annually 4300 cases of new mycobacterial infections are reported in Saudi Arabia [3]. There are several published case reports of extra pulmonary NTM diseases in the country. Most of these reports were focused on patients who underwent transplantations and particularly reported with peritonitis [4], [5], [6]. Nevertheless, only few studies analyzed the pulmonary NTM infections and very little is known about the NTM prevalence in the country [7], [8], [9]. Interestingly a novel species of mycobacteria (*M. riyadhense*) also reported from the country [10]. However the prevalence is expected to rise as immuno-compromised medical conditions (transplantations, genetic disorders, various immunosuppressive illness and treatments) are high. Moreover the treatment of NTM infections in the country is empirical as species identification facilities are limited to common species. There have been no nationwide studies so far in the Gulf Corporation Council countries (GCC) particularly in Saudi Arabia on the clinical relevance and species diversity of NTM. Therefore a prospective study to determine the species spectrum of both pulmonary and extra-pulmonary NTM isolates from all the provinces of the country was conducted. We report here for the first time the diversity of clinically relevant NTM in Saudi Arabia.

## Materials and Methods

### Study samples

During June 2009–July 2010, 2456 mycobacterial culture isolates were collected from tuberculosis reference laboratories which are the major TB diagnostic destinations in the country. The isolates were cultured in Lowenstein-Jensen's media and Mycobacterial Growth Indicator Tubes (MGIT 960; Becton Dickinson, NJ, USA). The genomic DNA was extracted by using the standard spin column technique (Qiagen, Hilden, Germany).

Primary NTM screening was carried by using the GenoType MTBC kit (Hain Life Science, Nehren, Germany) as per the manufacturer's recommendation. Resulting to the primary screening, 95 non repetitive isolates were characterized as NTM and subjected to further investigation. The demographical and clinical data of the patients were collected by referring to medical records and crosschecked against the national mycobacterial registry maintained by the Ministry of Health. All the data collected for the study was anonymized and no patient identifiers were used throughout data collection and analysis period. The study was approved by the research ethical committee of King Faisal Specialist Hospital and Research Centre.

### Identification of NTM species

Identification of species was carried out by the reverse hybridization based line probe assay according to the manufacturer's recommendations and procedure described elsewhere [11]. Primary identification used the kit GenoType Mycobacterium CM (Hain Life science, Nehren, Germany) which targets the most common species of mycobacteria. Unidentified isolates from this assay were further investigated with the kit for additional species GenoType Mycobacterium AS (Hain Life science, Nehren, Germany). The final results after hybridization on the strips were scanned using the automated system Genoscan (Hain Life science, Nehren, Germany).

### Data analysis

The results of hybridization were interpreted by using the Blotrix software (Hain Lifescience, Nehren, Germany). The clinical and demographical data were analyzed by using SPSS version 19.0 (IBM, NY, USA) software package. The standardized diagnostic criteria of ATS/IDSA were applied to determine the clinical relevance of respiratory isolates [1]. As per the recommendations sputum samples with multiple NTM isolation and smear positivity were considered to be clinically relevant. Bronchial lavage or Non respiratory specimens with one time smear positivity and isolation were also considered as relevant. From the available clinical records of the patients, major risk factors were abstracted and analyzed.

## Results

### Study population

Demographical data revealed that majority of patients were born in Saudi Arabia (62.1%). The median of age was 43 years and the age group >60 years (41%) was the highly infected group followed by 6–40 (29.5%), 41–59 (23.2%) and ≤5 years (6.3%) respectively. Proportion of male patients (55.8%) was higher than females. The higher rate of NTM infections were observed in the central region of the country followed by Western and Eastern provinces (Table 1).

**Table 1:** Summary of the study subjects.

Parameters	Sample Proportion (No/%)
**Nationality**	
Saudi	59(62.1)
Non Saudi	36(37.9)
**Age groups**	
<5 Years	6(6.3)
6–40	28(29.5)
41–59	22(23.2)
>60	39(41)
**Gender**	
Male	53(55.8)
Female	42(44.2)
**Geographic Distribution**	
Central	30(31.5)
East	26(27.4)
South	11(11.6)
West	28(29.5)
**Sample type**	
Sputum	52(54.7)
Bronchial Lavage/aspirate	21(22.1)
Lymphnode biopsy/aspirate	14(14.7)
Pus	5(5.3)
Urine	2(2.1)
Pleural fluid	1(1)

Pulmonary specimens were predominant [sputum 52(54.7%)/bronchial lavage/wash-21(22.1%)] followed by lymph node biopsy/aspirate-14(14.7%), pus 5(5.3%), urine 2(2.1%) and pleural fluid 1(1.0%). Pulmonary infections were observed in 73 (76.8%) patients while 22 (23.2%) were extrapulmonary sites (Table 1).

### Diversity of NTM

**Figure 1:**
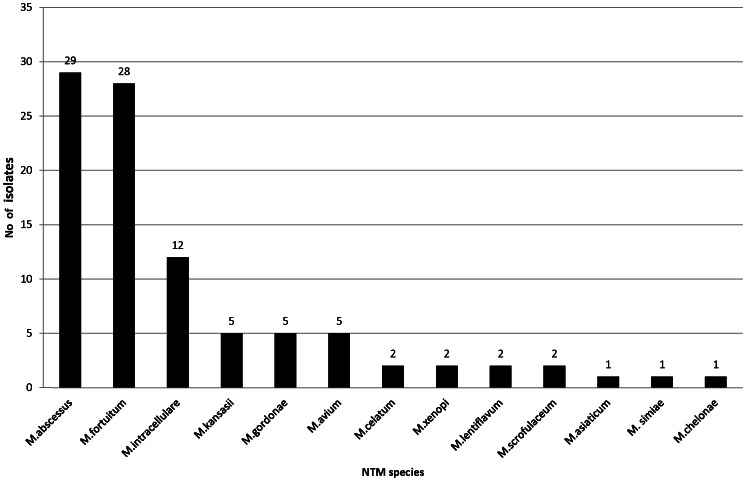
Overall species diversity of 95 clinical non-tuberculous mycobacterial isolates from Saudi Arabia. The diagram shows collective representation of pulmonary and extra-pulmonary isolates. The number of isolates with particular species are showed at the outer end of the bar diagram.

The spectrum of NTM species was high (13 species) and predominated by the rapid growing *M.abscessus* (30.5%) and *M.fortuitum* (29.5%). The slow growing species mainly consisted of *M. intracellulare* (12.6%), *M.kansasii* (6.8%) and *M.avium* (6.8%) respectively (Figure 1).

### Pulmonary and extra-pulmonary isolates

Among the 73 respiratory cases 49(67.1%) meet the ATS/IDSA guidelines for NTM disease, whereas 24 (32.9%) isolates were suspected as colonizers. *M.fortuitum* and *M. abscessus* were the most common respiratory pathogens and *M.fortuitum* also showed the highest colonization. *M.intracellulare, M.kansasii* and *M.avium* were also clinically relevant (Table 2). *M. abscessus* (36.4%) was predominant among the non respiratory isolates and interestingly *M.lentiflavum* and *M.scrofulaceum* were found only among extra-pulmonary cases (Table 3).

**Table 2:** Summary of the 73 pulmonary samples with NTM infections during 2009–2010 from Saudi Arabia.

Species	No/%	Specimen	AFB smear		Risk factors	No. of samples		Clinical Relevance*	
			Positive	Negative		Single	Multiple	Confirmed	Suspected Colonization
*M.fortuitum*	25(34.2)	Sputum BAL	16 6	3	PMTD† (4), HIV‡ (2), COPD± (3), CAPD++(2)	11 6	8	14	11
*M.abscessus*	21 (28.8)	Sputum BAL	10 7	3 1	PMTD(5), HIV(1), PFB¶	6 5	7 3	15	6
*M.intracellulare*	9(9.7)	Sputum	9		PMTD(2), COPD (2)	4	5	5	4
*M.kansasii*	5(6.8)	Sputum BAL	3 2		HIV(1), PMTD(1), CF# (1) COPD (1)	1 2	2	4	1
*M.avium*	5(6.8)	Sputum	5		HIV(1), CF(1), COPD(2)	2	3	3	2
*M.gordonae*	2(2.7)	Sputum	2		HIV(1)		2	2	
*M.xenopi*	2(2.7)	BAL	2		PMTD(2)		2	2	
*M.celatum*	2(2.7)	BAL	2		PMTD(1)		2	2	
*M.asiaticum*	1(1.4)	Sputum	1		PMTD		1	1	
*M. simiae*	1 (1.4)	BAL	1		PMTD		1	1	

* Based on ATS/IDSA 2007 guidelines.

† Previous mycobacterium tuberculosis disease.

‡ Human immunodeficiency virus.

¶ Pulmonary fibrosis.

# Cystic fibrosis.

± Chronic obstructive pulmonary disease.

++ Continuous ambulatory peritoneal dialysis.

**Table 3:** Summary of the 22 extra-pulmonary NTM infections observed in the study.

Mycobacterial species	No of cases/%	Source of specimen	Identified Risk Factors
*M.abscessus*	8(36.4)	LN (FNA/BP)†, pus	PMTD¶ (6)
*M.fortuitum*	3(13.6)	LN (FNA/BP), Pus, Pleural fluid	CF‡ (1), CAPD (1)
*M.intracellulare*	3(13.6)	LN (FNA/BP)	PMTD(2)
*M.gordonae*	3(13.6)	LN (FNA/BP)	HIV* (1), PMTD (1)
*M.lentiflavum*	2(9.1)	Urine	
*M.scrofulaceum*	2(9.1)	LN (FNA/BP)	PMTD
*M.chelonae*	1(4.5)	LN (FNA/BP)	PMTD

† Lymphnode/Fine Needle Aspiration/Biopsy.

¶ Previous mycobacterium tuberculosis disease.

‡ Cystic fibrosis.

* Human immunodeficiency virus.

### Risk factors

Suspected co-morbid conditions were available only for 51(53.7%) patients. Some of the patients had multiple risk conditions. Major predisposing conditions observed were, previous history of *M.tuberculosis* disease (PMTD) 28(29.5%), chronic obstructive pulmonary disorder (COPD) 11(11.6%), HIV reactivity-7(7.4%), continuous ambulatory peritoneal dialysis (CAPD) 5(5.3%), cystic fibrosis (CF) 3(3.2%) and pulmonary fibrosis 1(1%) respectively. Any other risk factors including the concomitant treatment or other immuno suppressive ailments among the study subjects were not available in the patient records (Tables 2–3).

## Discussion

This study sought to evaluate the diversity of clinically important NTM species in Saudi Arabia. According to recent reports the annual overall mycobacterial disease burden in Saudi Arabia is mainly observed among the migrant workforce [12]. However the current findings show an increasing problem of NTM infections among Saudis (62.1%). Predominance of the Saudi nationals and gender male in the study are in concordance with previous studies which showed that origin of patient and gender are risk factors for NTM infections [13], [14]. The interesting fact is that, the higher number of cases were found among elderly and their role as a demographical risk factor for NTM infection was also previously described [14], [15]. However it is assumed from the findings that Saudi nationals particularly men above 60 years can be a relatively high risk group for NTM infections in the country.

Rate of clinically relevant respiratory diseases after applying the stringent guidelines (ATS/IDSA) were found to be high in the study (67.1%). This is a serious event to report the high significance of pulmonary NTM infections in the Saudi Arabian society. It is worth to mention that higher compliance of pulmonary cases with the standard diagnostic criteria may either reflects the sampling limitation (small size and not complete coverage) or the real picture of NTM prevalence in the country. Nonetheless this indicates an emergency event which seriously requires attention from concerned authorities. Neighboring countries also reported an increasing prevalence of NTM diseases recently [13], [16].

Here we report for the first time the clinical relevance of six species namely *M. celatum, M. xenopi, M. scrofulceum, M. lentiflavum, M. asiaticum* and *M. simiae* in Saudi Arabia. However there were previously reported cases of *M. abscessus*, *M.fortuitum, M. kansasii, M.chelonae, M.gordonae, M.intracellulare and M.avium* [9], [17], [18], [19], [20].

Varghese et al. recently reported the predominance of *M.abscessus* in causing chronic lung infections among healthy individuals in the country [9]. The domination of the rapid growing mycobacterial species *M.fortuitum* and *M.abscessus* among pulmonary and extra-pulmonary cases in the current study are in concordance with a recent report from Eastern Asia [21]. The predominance of *M.fortuitum* in the country par resembles the recent finding from the neighboring country Kuwait [13]. The most common NTM species found in the Asian countries and rest of the world was *M. avium* complex. On the other hand, *M.intracellulare* sequevars were found in Saudi Arabia [21], [22]. Existence of *M. lentiflavum* in urine is an unusual site of infection that was found in two cases corroborating with a previous study from Greece with similar findings [23].

Preexisting pulmonary conditions were the highest risk factors found in the study and remaining patients may have concurrent illness or immunosuppressive medications. The regular NTM infections among CAPD patients in the country was reported in earlier studies which supports the current observations [24]. NTM diseases among healthy individuals is not rare as it has been reported from Saudi Arabia also [9]. In addition patients without known risk factors may possess a unique genetic susceptibility or environmental exposure to NTM [25]. The immunosuppressive conditions other than the respiratory illness including solid organ transplantation, hematological and other malignancies, chronic renal failure and diabetes mellitus are emerging challenges in the country [26], [27], [28]. In addition the increased prevalence of primary immuno deficiencies mainly because of the world's highest rate of consanguinity (∼60%) makes the Saudi population more vulnerable to NTM infections [29].

The study has certain limitations; the sample volume was smaller to conclude about the nationwide prevalence of NTM, information of concomitant medications and malignancies were not available for any studied cases in the referred records. On the other hand the study was not designed for a nationwide 100% sample collection. Due to minimal representation of isolates to each geographic region a prevalence estimate preparation was impossible.

In conclusion the magnitude of true NTM disease in Saudi Arabia is escalating. Presence of highly diverse NTM species even among immunocompetent individuals needed an immediate attention. The emergence of rapid growing species with predominance shows an opposite trend to the Western world where slow growing species are dominated. This difference might be due to the subtropical or desert like geographical status of the country. We emphasize on the pathogenic potential of rapid growing NTM species to cause pulmonary and extra-pulmonary diseases in the Saudi Arabian community. This study warrants the need to explore all the risk factors that lead to the NTM disease in the country. Thus the findings demand a large scale nationwide study in collaboration with neighboring countries to find the real magnitude of NTM prevalence in the Arabian Peninsula.
